# Two-dimensional honeycomb network through sequence-controlled self-assembly of oligopeptides

**DOI:** 10.1038/ncomms10335

**Published:** 2016-01-12

**Authors:** Sabine Abb, Ludger Harnau, Rico Gutzler, Stephan Rauschenbach, Klaus Kern

**Affiliations:** 1 Max Planck Institute for Solid State Research, Heisenbergstraße 1, 70569 Stuttgart, Germany; 2 Bernhäuserstraße 75, 70771 Leinfelden-Echterdingen, Germany; 3 Institut de Physique de la Matière Condensée, Ecole Polytechnique Fédérale de Lausanne, CH-1015 Lausanne, Switzerland

## Abstract

The sequence of a peptide programs its self-assembly and hence the expression of specific properties through non-covalent interactions. A large variety of peptide nanostructures has been designed employing different aspects of these non-covalent interactions, such as dispersive interactions, hydrogen bonding or ionic interactions. Here we demonstrate the sequence-controlled fabrication of molecular nanostructures using peptides as bio-organic building blocks for two-dimensional (2D) self-assembly. Scanning tunnelling microscopy reveals changes from compact or linear assemblies (angiotensin I) to long-range ordered, chiral honeycomb networks (angiotensin II) as a result of removal of steric hindrance by sequence modification. Guided by our observations, molecular dynamic simulations yield atomistic models for the elucidation of interpeptide-binding motifs. This new approach to 2D self-assembly on surfaces grants insight at the atomic level that will enable the use of oligo- and polypeptides as large, multi-functional bio-organic building blocks, and opens a new route towards rationally designed, bio-inspired surfaces.

## Abstract (web summary)

Peptide nanostructures are currently arousing interest thanks to their potential applications in medicine, electronics and coatings. Here, through experiment and theory, the authors demonstrate exquisite control over surface peptide assembly behaviour through manipulation of amino acid sequence.

## Graphical abstract

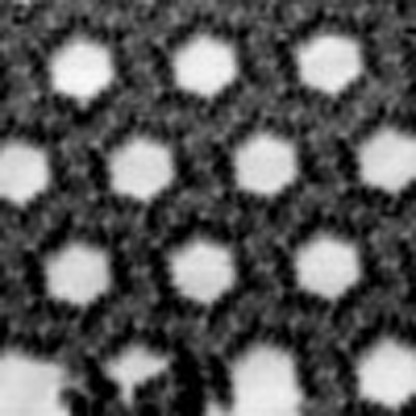

Inspired by nature, the engineering of versatile biomolecular nanostructures has been investigated extensively, demonstrating manifold possibilities for self-assembly in solution^1^,^2^,^3^ and in vacuum^4^. A rich variety of materials can be fabricated with outstanding properties for applications in fields ranging from energy storage and coatings to medicine^5^,^6^,^7^,^8^,^9^. The conceptual design and controlled manufacturing of biomolecular nanostructures programmed by the sequence was pioneered with DNA^10^. This supramolecular synthesis scheme is based on the specific molecular recognition of complementary base pairing in matching DNA strands. Single-stranded DNA sequences composed of the four nucleic acids are defined such that they produce various three-dimensional nanostructures when they bind to their complementary strands^11^. In biological systems, DNA is mainly intended for information storage, thus is highly stable, but lacks flexibility and catalytic versatility.

In contrast to DNA, peptides and proteins, which are programmed by their amino-acid sequence, exhibit an adaptive structure and a sequence-specific function. Peptides are attractive as building blocks in nanotechnology because they inherently provide chemical versatility and conformational adaptability combined with biodegradability and a facile synthesis for almost arbitrary sequences. Synthetically engineered peptide nanostructures can be very robust^12^ and exhibit interesting properties such as semiconductivity, luminescence or high mechanical rigidity^13^,^14^.

While the combinatorial space achieved by sequences of the 20 canonical amino acids offers an enormous playground, it also impedes a straight-forward prediction of self-assembled structures by sequence alone. So far, the formation of dipeptide nanostructures has been investigated thoroughly demonstrating, for instance, that aromatic moieties form an effective zipper-like-binding motif for the formation of nanotubes^13^. Several other approaches that also apply to longer sequences^5^,^6^,^15^ were introduced such as ionic self-complementary peptide sequences for the formation of membranes^16^ or amphiphilic-building blocks^17^. Recently, Frederix *et al.*^18^ have mapped out the complete sequence space for tripeptides in neutral, aqueous solution finding a general agreement of the sequence-dependent assembly behaviour with the results of the above-mentioned approaches. The complete mapping of the sequence space may be possible for very short sequences, nonetheless, there is a need for ubiquitous rules for rational design that apply to polypeptides in general.

Atomistic detail is pivotal for the development of comprehensive models, offering a thorough understanding that may ultimately lead to establish design principles. Subnanometre resolution in real space can be achieved by high-resolution scanning probe microscopy, such as atomic force microscopy or scanning tunnelling microscopy (STM). STM studies on the assembly of small organic molecules demonstrate that the concepts of supramolecular chemistry can be transferred to molecules on surfaces in ultra-high vacuum (UHV): on the basis of high-resolution imaging, the engineering of nanostructures from small molecules, formed due to intra- and intermolecular interactions including hydrogen bonding, polar interactions and van-der-Waals interactions, is very well understood down to the single molecule level^19^,^20^. Moreover, STM investigations of short peptides adsorbed on surfaces demonstrated adaptive binding^21^, as well as conformational flexibility relevant for cooperative fibril-like growth^22^.

Due to the non-volatile character of polypetides, which emerges with increasing sequence length, only very short peptides (of up to four amino acids) can be evaporated to prepare UHV samples compatible with STM investigations. This limitation in size particularly restricts the complexity and variability of the building blocks, and thus most studies present assemblies of small peptides and focus on amyloid-forming motifs such as β-sheet-like structures^22^,^23^,^24^. To overcome this size limitation, we apply soft-landing electrospray ion beam deposition (ES-IBD), because the generation of intact gas-phase ions by electrospray ionization is not limited by low thermal stability^25^,^26^,^27^. The deceleration of the molecular ions for soft landing avoids fragmentation and ensures that the molecules are intact after deposition. Thereby, we ensure the intact and highly pure deposition of the selected species in UHV via mass spectrometry, mass filtering and soft landing, features intrinsic to ES-IBD^25^,^28^. The high quality and purity of the fabricated surfaces is essential to the subsequent high-resolution structural analysis by STM and its interpretation. We demonstrated the capacity of STM/ES-IBD in previous works for the proteins cytochrome *c* and bovine serum albumin, in which we show that the structural details of individual, large polypeptides can be resolved by STM with sub-nanometre resolution^29^. However, we found that for these long amino-acid sequences, the polymer properties dominate over intra- and intermolecular interactions and no rational nanostructure formation was possible^30^.

Here we employ oligopeptides angiotensin I (At-I) and angiotensin II (At-II) as bio-organic building blocks for two-dimensional (2D) assembly on Au(111). Combining ES-IBD, high-resolution STM imaging and atomistic modelling, that is, molecular dynamics (MD) and density functional theory (DFT), allows the role of each amino acid in the individual peptide to be addressed. Morphology, symmetry and structural details are resolved by STM at subnanometre level as convolution of local density of states with topography. The experimental data gives a unique fingerprint of the molecular assembly and thereby provides strong constraints for the MD model of the assemblies. Finally, the validity of the MD-optimized structure is tested by comparing simulated STM images of that MD structure with the measured STM images. We note that only by this compound approach we are able to provide a rigorous identification and rationalization of the complex binding motifs (for more details, see Supplementary Methods). On the basis of highly detailed insight into the binding motifs, we can suggest a mechanism and understand the effect of sequence manipulation on the self-assembly. We demonstrate this capability by manipulating the sequence from At-I to At-II to achieve long-range ordered hexagonal networks. With this approach, it will be possible to understand the complex self-assembly of oligo- or even small polypeptides at a level that allows rational sequence-controlled fabrication of molecular nanostructures employing peptides to be established.

## Results

### Self-assembly of angiotensin I on Au(111)

**Figure 1:**
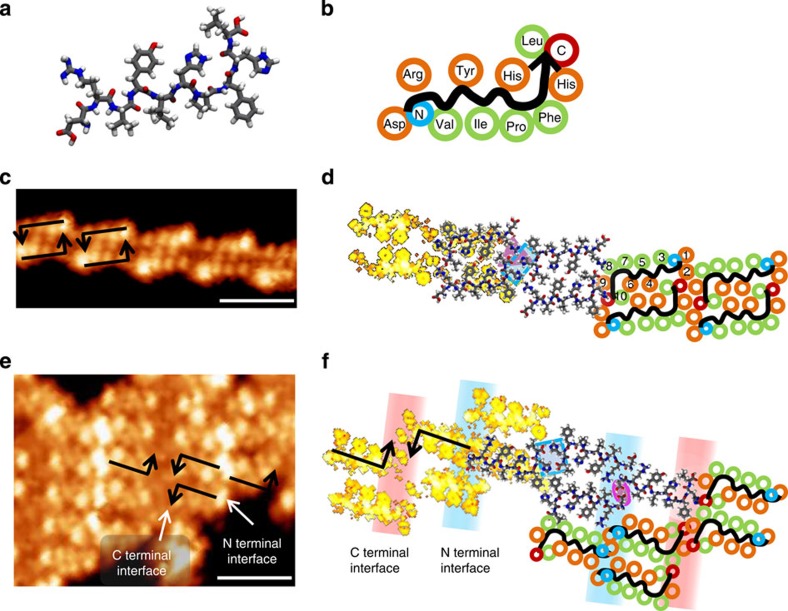
Decapeptide angiotensin I on Au(111). (**a**) MD-optimized molecular structure of adsorbed At-I. (**b**) Scheme of the molecular structure of At-I with the residues highlighted in orange (polar) and green (nonpolar). (**c**) STM image (1.5 V, 29 pA; scale bar, 4 nm) of structure A with schematic model of the staggered chains assembly. (**d**) Atomistic MD model of structure A with highlighted regions (magenta and blue) indicating important binding motifs. On the left side, the simulated STM image of that MD structure is overlaid. On the right side, a schematic model can be found. (**e**) STM image (1.5 V, 29 pA; scale bar, 4 nm) of structure B with schematic model of the stacked assembly and indication of rows of alternating contrast at the C and N terminal interface. (**f**) Atomistic MD model of structure B with highlighted regions (blue rectangle and magenta oval) indicating important binding motifs and the region of the C and N terminal interface. On the left side, the simulated STM image of that MD structure is overlaid. On the right side, a schematic model can be found.

Bridging the gap between proteins and small peptides, we choose At-I (DRVYIHPFHL, *M*=1,296 g mol^−1^, Fig. 1a,b) to explore rational design principles of peptide assemblies on surfaces. At-I is an ideal starting point because its sequence is dominated by sterically demanding groups, resulting in a rigid conformation of little variability if confined to two dimensions by adsorption to a surface. The sequence offers a variety of residues that promote non-covalent interactions. Atomistic MD simulations of the landing process of At-I on the Au(111) surface yield a structure in which the peptide backbone has an L-shape (Fig. 1a). Similar to a β-strand, the chemical moieties of the amino acids are located alternatingly on both sides of the backbone resulting in a near-complete segregation of polar (orange) and nonpolar (green) moieties, as illustrated in Fig. 1b.

After deposition of At-I on the Au(111) surface and cool down to 40 K, we observe two kinds of ordered assemblies by STM (see Supplementary Fig. 1 for overview images). Structure A (Fig. 1c) consists of chains of staggered At-I dimers that are ∼2.3 nm wide and 2.7 nm long. From the size and the apparent C_2_ symmetry as well as defect structures (Supplementary Fig. 2), we conclude that two peptides are aligned in an antiparallel manner, forming the dimer as indicated in Fig. 1c by the black arrows, which follow the backbone from N to C terminal.

Atomistic MD modelling of the dimer chain as shown in Fig. 1d reveals that the nonpolar residues (Val3, Ile5, Pro7 and Phe8) lie on the outside of the assembly. Due to the L-shape of the backbone, the polar moieties of the peptides within the dimer cannot come into close proximity. Thus, the polar groups in the inside of the assembly (Tyr4 and His6) do not form hydrogen bonds but stabilize the dimer by weaker polar interactions. Hydrogen bonds are only formed between the C terminal and the Arg2 moiety. This formation also interacts with the His9 residue of the neighbouring dimer, indicated by the magenta triangle in Fig. 1d. In addition, the His9 residues of two adjacent dimers connect diagonally, shown by the blue rectangle. This motif is encapsulated by the phenyl rings of Phe8, which passivates the polar groups from the outside. In combination, the self-passivation on the long side and the binding motif at the short ends lead to the formation of the staggered dimer chain. This assignment is supported by the simulated STM image of the MD structure that qualitatively reproduces the experimental data. A more detailed comparison can be found in the Supplementary Note 1.

Structure B is imaged as a compact assembly in which the At-I molecules are stacked parallel along the long side forming rows of 3 nm width (Fig. 1e) with alternating contrast at the interface of rows as indicated in Fig. 1e. Adjacent rows are rotated by 180°, thus interacting alternatingly via the C terminal or the N terminal. Using these observations as constraints for the MD model yields a structure shown in Fig. 1f. Here the Asp1 residues at the N terminal form hydrogen bonds with each other (magenta oval in Fig. 1f), while the C terminal binds to the His9 of an adjacent peptide (blue rectangle). However, a clear assignment of the structure and the type of the interface cannot be achieved by the MD structure alone. With the simulated STM image, we can reproduce the alternating intensity at the C- and N-terminal interfaces and thus unequivocally assign the interfaces. A detailed discussion of the simulated STM image and a comparison with the experimental data is offered in Supplementary Figs 2 and 3 and Supplementary Note 1.

### Manipulating the amino-acid sequence to steer self-assembly

In both assemblies A and B, the monomer in the STM images resembles an arrangement of four major protrusions forming a line and a fifth protrusion in second row. The similar shape of the building block, in which the protrusions only differ in contrast, is indicative of the peptide backbone having little conformational freedom, while the moieties can adapt to different conformations. An L-shape of the backbone is found in the MD-optimized structures for both types of assemblies. The simulated STM image in Fig. 1d,f, that is, a convolution of the electron density and topography, which is based on the MD structure, is in good agreement with the appearance of the molecular assembly in the STM images. In general, the linear appearance of the molecule in STM arises because the residue of Asp1 and the C terminal with Leu10 do not carry appreciable electron density and thus do not contribute to the signal.

The L-shaped backbone causes steric hindrance at the long side of the molecule that contains most of the polar residues. These polar groups are denied close contact to the neighbouring molecule and thus cannot form hydrogen bonds. Instead, the interactions that lead to long-range order have to take place at the residues close to the peptide terminals. In particular, His9 and the C terminal are involved in the intermolecular connection of both types of assemblies, however in different arrangements (blue rectangles in Fig. 1d,f). This shows that they do not provide a specific motif to yield one dominant, long-range ordered structure.

**Figure 2:**
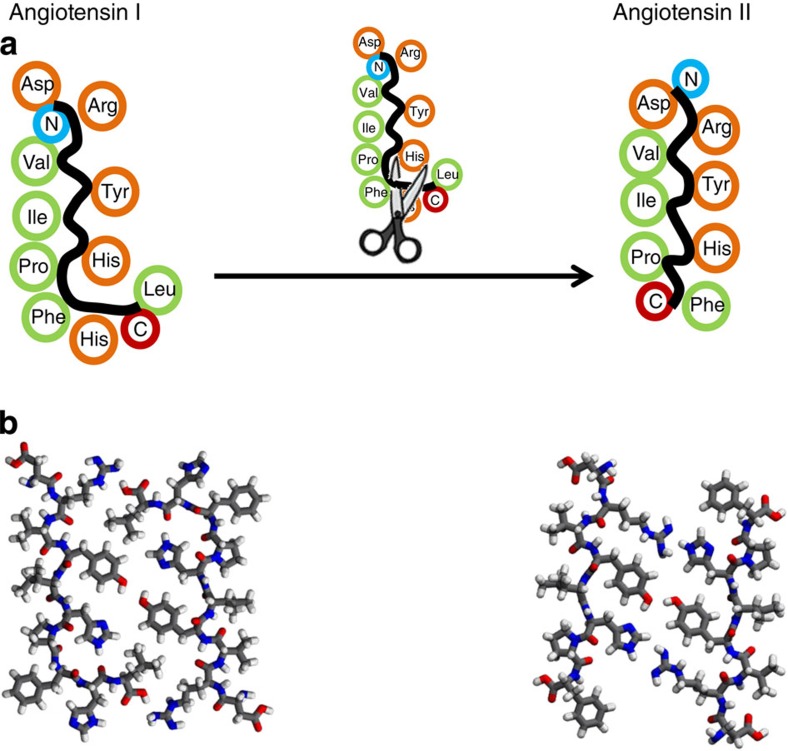
Sequence manipulation from angiotensin I to angiotensin II. (**a**) Schematic of the removal of His9 and Leu10 at the C terminal converting the L-shaped backbone of At-I to a linear backbone in At-II on the surface. The segregation in polar (orange) and nonpolar (green) residues is maintained. (**b**) MD-optimized structures of the dimers on the surface.

To enforce one stable, well-ordered assembly at the surface, we manipulate the peptide sequence by the removal of two amino acids (His9 and Leu10) at the C terminal, as depicted in Fig. 2a. This results in the eight amino-acid peptide At-II (DRVYIHPF, *M*=1,046 g mol^−1^), the biological successor of At-I with a length of 2.3 nm and a new C terminal at the Phe8. This modification removes the steric hindrance and thus stabilizes the dimer formation. In At-II, a straight backbone configuration promotes the close proximity of the polar groups inside, enabling hydrogen bonding. In addition, the Arg2 residue can now take part in the binding at the position of the removed Leu10 moiety. Each dimer is thus stabilized on the inside by three polar interactions; Arg2-His6′, Tyr4-Tyr4′, His6-Arg2′ (Fig. 2b), while the long sides of the dimer are still passivated by the nonpolar alkyl residues (Val3, Ile5 and Pro7). Subsequent assembly of the dimers can take place due to the unpassivated C and N terminals, as well as other polar (Asp1) and nonpolar (Phe8) residues.

### Self-assembly of angiotensin II into honeycomb networks

**Figure 3:**
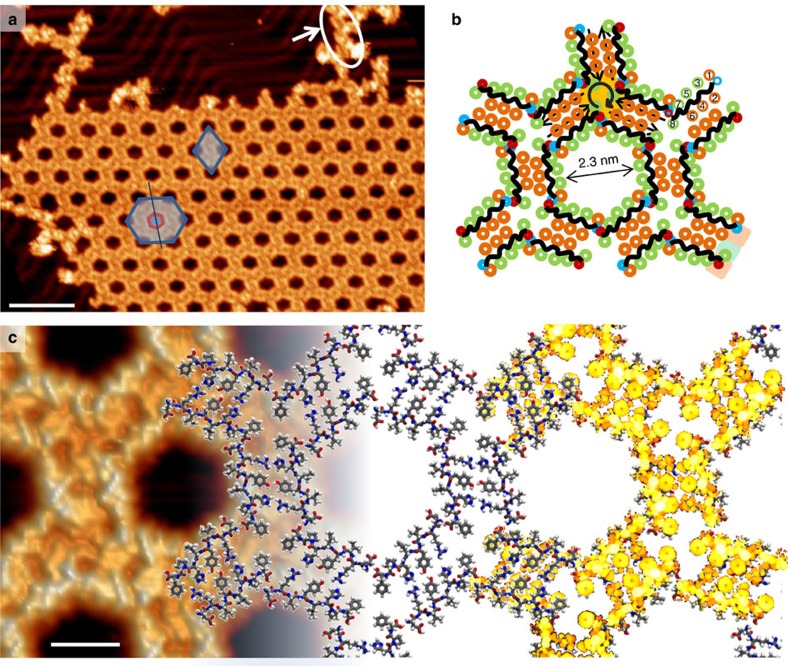
Network of angiotensin II on Au(111). (**a**) STM image (1.3 V, 35.5 pA; scale bar, 15 nm) of a large, well-ordered honeycomb network. The diamond indicates the unit cell, the hexagon demonstrates the chirality of the network as the hexagonal pore (red) is tilted anticlockwise by 6° with respect to the hexagonal superstructure (blue). In the upper right corner, a linear arrangement is encircled in white. (**b**) The schematic arrangement of the At-II molecules according to MD simulations demonstrates the nonpolar (green) decoration of the pore with the polar residues (orange) in the inside of the nanostructure. A circular arrow indicates the chiral vertex. The polar–nonpolar–polar-binding motif at the short edge of the dimer is indicated by a green/orange background at the lower right corner. (**c**) High-resolution STM image (1.3 V, 35.5 pA; scale bar, 2 nm; left) overlapped with the MD-simulated model (middle) and the calculated electron density (right) for comparison with the STM image. DFT was performed on the optimized structure of a dimer and subsequently overlaid with the MD structure.

After the deposition of At-II, we find a significantly different morphology compared with the At-I. Instead of small assemblies, we observe extended domains of a honeycomb network by STM (see Fig. 3a and survey images, Supplementary Fig. 4). The network exhibits large hexagonal pores with an inner diameter of 2.3 nm (4.5-nm^2^ pore area). The unit cell of the structure accounts to *a*=*b*=5.5 nm at an angle of 120°. The network is chiral, which is manifested in the rotation of the hexagonal pores with respect to the peptide lattice's principle directions anticlockwise by 6° (Fig. 3a). Remarkably, almost all peptides exhibit a uniform adsorption geometry within the network despite being flexible and multi-functional.

This specific symmetry of the network in combination with the clearly observable double-walled nature of the network as seen in Fig. 3c provides good initial guesses for the MD simulation of the network. The MD-optimized molecular structure presented in Fig. 3c fits best to our observations and represents a stable, lowest-energy configuration among many candidate structures. In this model, the vertex is formed by the interaction of the C terminal of one peptide backbone with the N terminal in an adjacent dimer (Fig. 3b). At the same time, the circumference of each pore is almost exclusively decorated by nonpolar groups, passivating the assembly.

**Figure 4:**
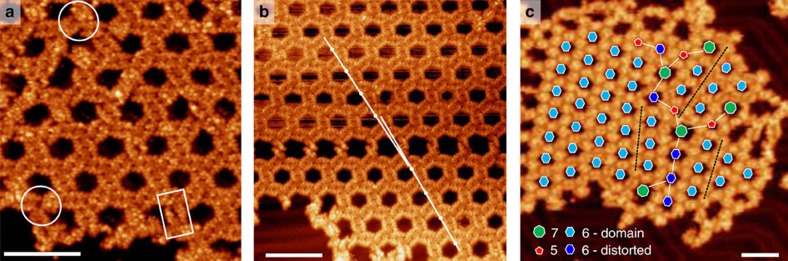
Defects of the honeycomb network. (**a**) STM image (1.8 V, 32.6 pA; scale bar, 10 nm) showing distance defects within the dimer (white rectangle) and rotational defects at the vertex (white circle). (**b**) STM image (1.3 V, 35.5 pA; scale bar, 10 nm) of a linear grain boundary running horizontally in the central part of the image. The domains are tilted by ∼1° indicated by the white lines. (**c**) STM image (2.4 V, 38.7 pA; scale bar, 10 nm) of grain boundaries with larger rotational offset (∼16–25°) resulting in grain boundaries with seven-membered (green) and five-membered (red) rings to reduce stress.

On the basis of the high-resolution observations of the network (Fig. 3c) and its defects (Fig. 4; Supplementary Fig. 5; Supplementary Note 2), we can validate the molecular arrangement that is simulated by MD (for large-area network simulation see Supplementary Fig. 6). In addition to the matching symmetry and dimensions of the model, the simulated STM image of the MD-optimized structure of a dimer reproduces the characteristic features of the recorded STM images. The highest contrast is observed at the peptide backbone, which is represented by an intense ridge at the edge of the pores. In addition, the Tyr4 residue and His6 residue exhibit the highest signal within the dimer arrangement, giving rise to distinct features in the high-resolution STM image. Most convincingly, the three phenyl groups are the only features that exhibit electron density at the vertex, leading to three characteristic protrusions accompanied by three depressions forming a triangle—a unique fingerprint of the vertex motive clearly seen in the STM image in Fig. 3c (Supplementary Fig. 7).

Staggered linear arrangements of dimers are occasionally observed at the edges of the islands, indicated for instance in Fig. 3a in the upper right corner. From the model, we see that a polar–nonpolar–polar-binding motif is formed at the ends of the dimer, involving the C-terminal and the Phe8 residue of one peptide, and the Asp1′ residue of the other peptide. This motif promotes the staggered linear arrangements, which offer a binding site to add a third dimer and form the vertex. The Asp1 residue still remains present at the rim of the pore, however the angled arrangement of the two adjacent dimers sterically hinders the approach of another peptide or dimer.

While the threefold symmetry of the vertex might be supported by the substrate symmetry, it is also a direct consequence of the free energy of the system. Coulomb and van-der-Waals interactions have more impact due to the packing of dimers into hexamers. This vertex structure is energetically more favourable for instance over a linear chain arrangement with the same amount of possible hydrogen bonds between the dimers because it is more compact and thus more groups can interact with each other.

Despite the complex and specific binding of the peptides, the honeycomb network demonstrates a high fault tolerance. The position and arrangement of the molecules in the network are clearly revealed by the zero-dimensional defects shown in Fig. 4a. The distance between the two peptides is increased in the dimer that is highlighted by a white rectangle, making the individual molecules distinguishable. This observation confirms the antiparallel arrangement in a double-walled honeycomb network. Furthermore, at the nodes, the binding of three dimers can be askew, marked with white circles in Fig. 4a. This defect directly reveals the chiral, interlocked assembly.

Figure 4b shows a linear grain boundary between two domains that are slightly rotated by approximately 1° against each other. At this angle, the width of the grain boundary increases by 8 Å per hexagonal pore. In the STM images, the appearance of the dimer motif changes along the defect as it is stretched to compensate for the stress. The incorporation of this line defect also requires a rotational variability of the binding at the vertex motif, which is visible in the STM topography.

If the rotation of the domains is much larger, in the order of 16−25° as in Fig. 4c, a new regime of fault-compensation appears. In such a case, the stress is reduced by forming a grain boundary of seven- and five-membered pores, a fundamental scheme often found in systems of similar geometry independent of the length scale, for instance in graphene^31^ or in curved colloidal 2D crystals^32^. Again, the formation of this defect is only possible due to the tolerance of the At-II vertex motif with respect to the alignment and rotation of the dimers.

The rotational orientation of the At-II domains appears to be unrestricted by the underlying Au(111) lattice as many different relative orientations are observed. This suggests that the peptide–peptide intermolecular interaction is dominating over the binding to the substrate. The Au(111) substrate provides 2D confinement but does not impose a specific substrate-superstructure relation.

While most work to estimate the peptide–substrate interactions has been performed for individual amino acids^33^,^34^,^35^, the systems considered here deal with complete oligopeptides. A representative discussion about the interaction of an amino acid with the substrate is offered in the Supplementary Methods, referring to Supplementary Tables 1-4 and Supplementary Fig. 8. Due to the bulky residues, the strong peptide bonds and the restriction in two dimensions, the molecule on the surface is relatively rigid. Therefore, the different affinities of individual amino acids level out and the binding of the peptide must be considered as a compound effect involving entire molecules rather than the binding of the individual amino acids.

## Discussion

The amino-acid sequence in naturally occurring proteins controls the conformation, as well as the specific interactions of the proteins. Here we demonstrate a remarkably similar scheme, by which oligopeptides form long-range ordered nanostructures in two dimensions at a surface despite the apparent complexity and flexibility of the molecular building block. On the basis of the submolecular STM resolution that validates the atomistic MD simulations, we are able to obtain detailed models that allow for the identification of basic design principles and binding motifs.

By comparing the self-assembly of At-I and At-II, which only differ by two amino acids, we learned that using sterically demanding amino acids avoids backfolding and self-passivation of the single peptides on the surfaces resulting in a rigid building block of predictable behaviour. In addition, a balanced distribution of polar and nonpolar moieties is essential to provide specific binding sites, as well as passivation of the assembly, respectively, which is crucial for the long-range order assembly of oligopeptides on surfaces.

Our approach of obtaining a microscopic understanding from highly resolved STM measurements requires the feedback with MD and DFT modelling, and achieves for the first time the sequence-programmed assembly of oligopeptides on surfaces at the amino-acid level. The detailed experimental mapping of peptides and peptide assemblies on surfaces will serve as a model system to validate the emerging theoretical approaches in this field^30^,^34^,^36^. Together, high-resolution STM data and atomistic simulations allow for the exploration of promising binding motifs and their rational optimization.

## Methods

### Experiments

High-purity peptides At-I and II were acquired from Sigma Aldrich (A9650 and A9525, respectively). Solutions for electrospray ionization were prepared by dissolving the peptide in a water–ethanol 1:1 mixture with ∼0.1% formic acid to result in a concentration of 10^−4^ M. These solutions were used with our home-build ES-IBD (Supplementary Fig. 9; Supplementary Methods) to bring the molecules into the gas phase.

To ensure high-quality samples, the species was mass selected before deposition (Supplementary Fig. 10). For deposition, the ions were decelerated to 10 eV to ensure soft landing of the biomolecules. In this energy range, fragmentation of the molecular ions upon deposition can be neglected. During the deposition, the sample was held at room temperature and the ion current was measured on the sample to estimate the coverage (At-II, *z*=+2, 43 pAh; At-I, *z*=+3, 60 pAh; pAh=picoampere hours).

Before the deposition, a Au(111) single crystal was cleaned by subsequent cycles of sputtering (1 kV, 30 min) and annealing (930 K, 10 min) and then transferred into the deposition chamber without breaking the vacuum. After deposition, the sample was transferred to the STM chamber into a variable temperature STM (Omicron VT-STM). STM measurements were conducted at 40 K to immobilize the adsorbents on the surface. Measurement parameters are given with respect to the grounded sample. STM images were processed with WSxM 5.0 (ref. 37).

### Theory

All MD simulations were performed using the GROMACS software^38^ together with the OPLS-AA force field^39^. The energy function for the simulations comprises bonded interactions such as bond stretch, bond angle and dihedral terms, as well as non-bonded interactions described by Coulomb and van-der-Waals potentials. Moreover, the positions of the gold atoms were frozen at their crystallographic values. To take into account the interaction between the gold atoms and the peptide, the force field was extended by using the GoIP force field^40^. For simulating the landing process, the protonated peptides were transported to the gold surface under the influence of an external electric field similar to our earlier study on the deposition of proteins^30^. This led to quasi-2D conformations. Subsequently, the assembly was modelled starting with initial guesses based on the found adsorption conformations, as well as taking into account the symmetry, morphology and defects observed in the experimental data. A detailed description of the simulation of the landing process, as well as the modelling of the assembly can be found in the Supplementary Methods. For visualization of the molecular structures, VMD was employed^41^.

Orca 3.0 (ref. 42) was used for DFT calculations with the B3LYP functional and the def2-SVP basis set. The geometries of At-I and At-II were taken from MD simulations and the electronic structure was calculated without further geometry optimization. Furthermore, the metal substrate was not taken into account in the DFT calculations. For comparison with STM, the squares of all occupied molecular orbitals from the highest energy occupied molecular orbital to the molecular orbit within 2 eV from the highest energy occupied molecular orbital were summed up. The isosurface of this sum was convoluted with the relative distance away from the Au(111) substrate. Bright features (bright yellow) in the presented STM simulations correspond to molecular groups with high electron density and larger distance from the substrate than darker features (orange).

## Additional information

**How to cite this article:** Abb, S. *et al.* Two-dimensional honeycomb network through sequence-controlled self-assembly of oligopeptides. *Nat. Commun.* 7:10335 doi: 10.1038/ncomms10335 (2016).

## Supplementary Material

Supplementary Information Supplementary Figures 1-10, Supplementary Tables 1-4, Supplementary Notes 1-2, Supplementary Methods and Supplementary References
