# Supplementary material for: Two-dimensional honeycomb network through sequence-controlled self-assembly of oligopeptides
Source: Nat Commun. 2016 Jan 12;7:10335. doi: 10.1038/ncomms10335 (PMC4729956; doi:10.1038/ncomms10335)
Supplement: Supplementary Information — Supplementary Figures 1-10, Supplementary Tables 1-4, Supplementary Notes 1-2, Supplementary Methods and Supplementary References [file ncomms10335-s1.pdf]

Supplementary Figures

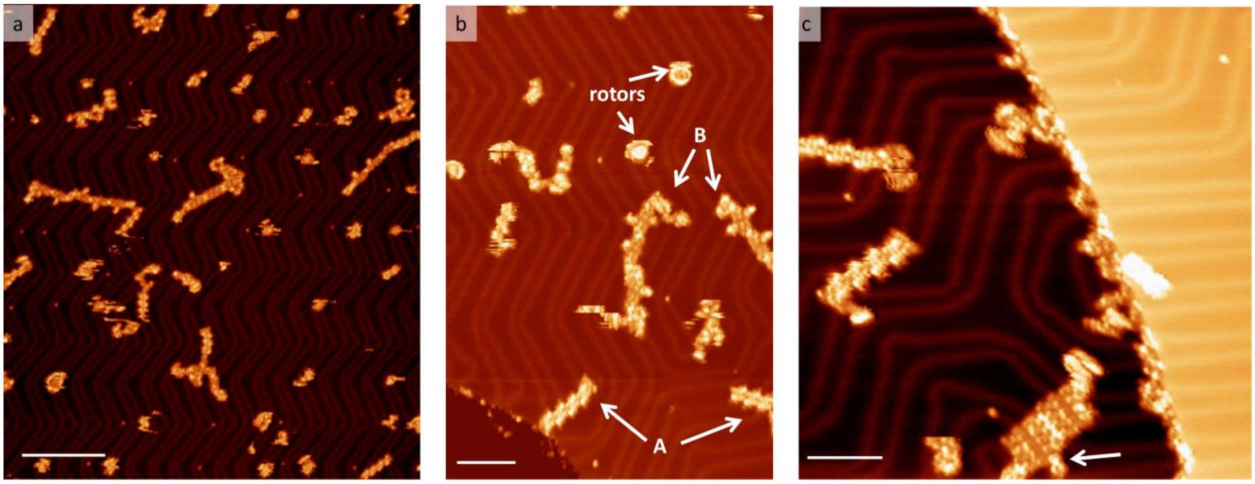

Supplementary Figure 1: STM images of At-I at 40 K. a, Overview STM image (1.5 V, 29 pA, scale bar 30 nm). **b,** STM image (scale bar 10 nm) in which structure **A** and **B** are observed along rotating molecules pinned to the elbow sites. At structure B additionally attached molecules are found at the rows of low STM intensity. **c,** STM image (scale bar 10 nm) in which both structure types are observed. The arrow points to an additional molecule adsorbed to structure **B** at the row of low contrast.

a

b

c

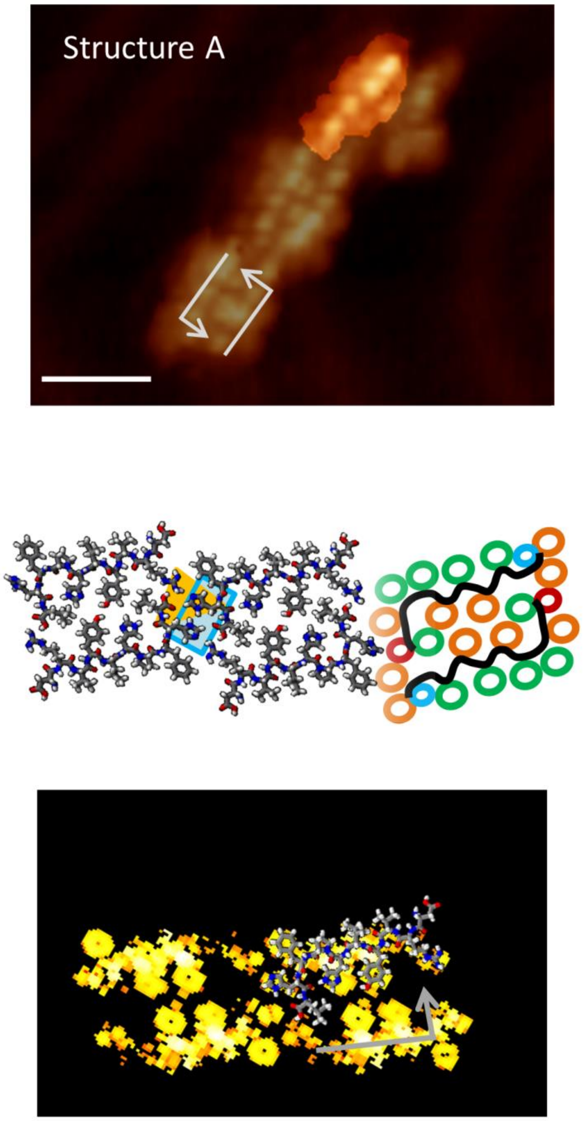

Supplementary Figure 2: Structural assignment of structure A of At-I. a, STM image (1.6 V, 29 pA, scale bar 3 nm) of structure A. A single peptide at the defect is highlighted **b,** MD optimized structure as described in Fig. 1d. **c,** simulated STM image at 2 V with an overlay of a single peptide. The grey arrows indicate the position of the peptide backbone.

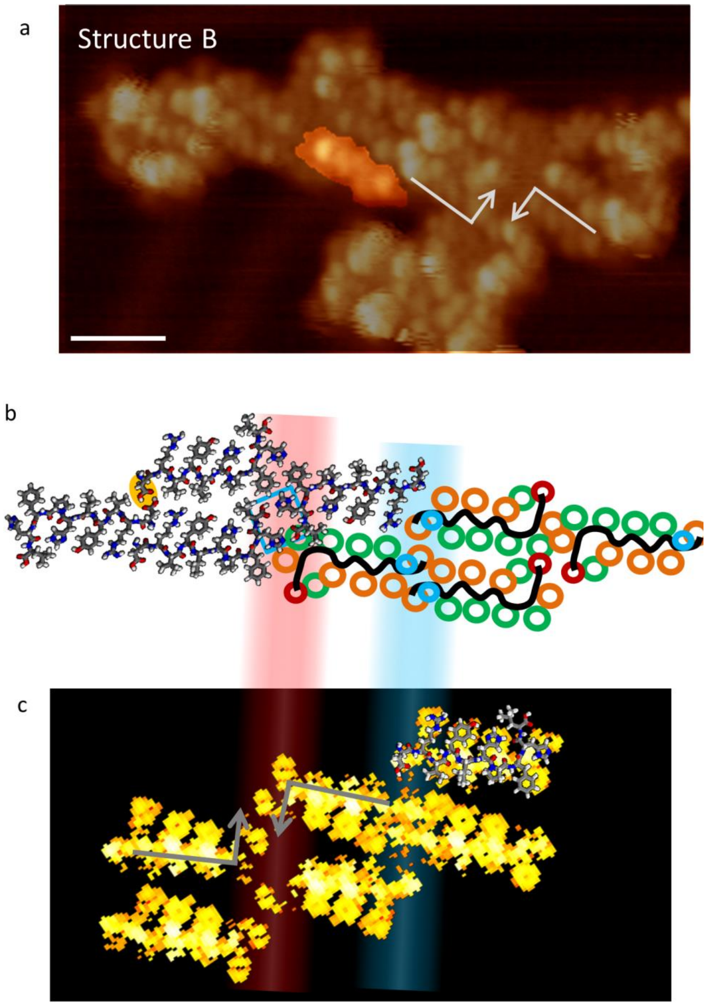

Supplementary Figure 3: Structural assignment of structure B of At-I. a, STM image (1.4 V, 29 pA, scale bar 3 nm) of structure B. A monomer is highlighted and the white arrows indicate the position of other peptides. **b,** MD optimized structure as described in Fig. 1f. **c,** simulated STM image at 2.5 V with an overlay of a single peptide. The grey arrows indicate the position of the peptide backbone.

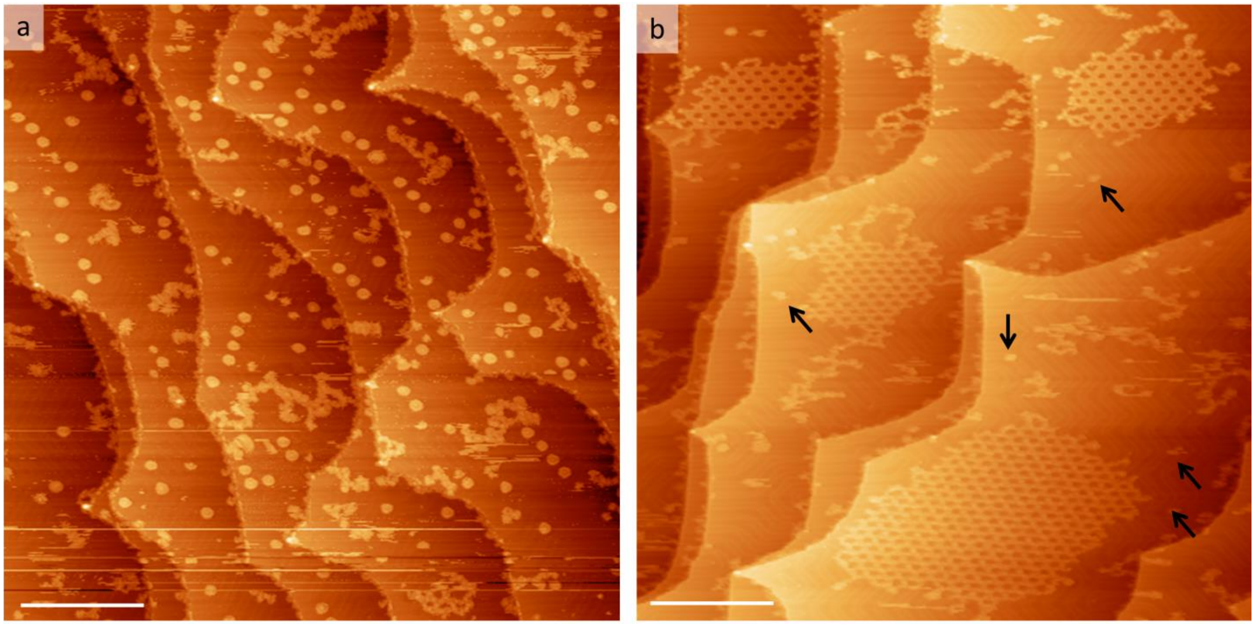

Supplementary Figure 4: STM images of At-II at 40K. a, Overview STM image (1.9 V bias, 44 pA tunnel current, scale bar 50 nm) on a terrace during cool down. The molecules are still mobile and mostly rotating molecules can be found. **b,** Overview STM image (1.3 V, 36 pA, scale bar 45 nm) at 40K: porous networks are formed nearly exclusively. Domain growth takes place on terraces rather than a step edge as the rotating molecules (some are indicated by an arrow) might be the seed for the assembly.

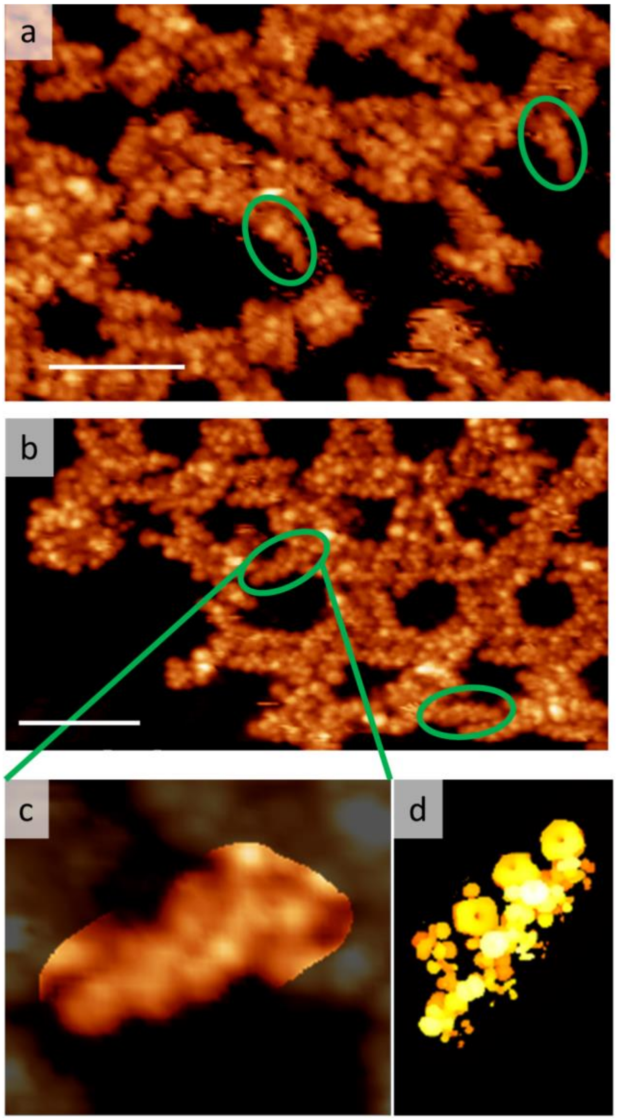

Supplementary Figure 5: Structural assignment of At-II. a, STM image (1.5 V, 29 pA, scale bar 6 nm) of the network and defects. The green circles highlight single peptides **b,** STM image (scale bar 6 nm) of a single peptide stabilized within the network. **c,** Magnification of **b** with a single peptide highlighted. **d,** simulated STM image at 2 V.

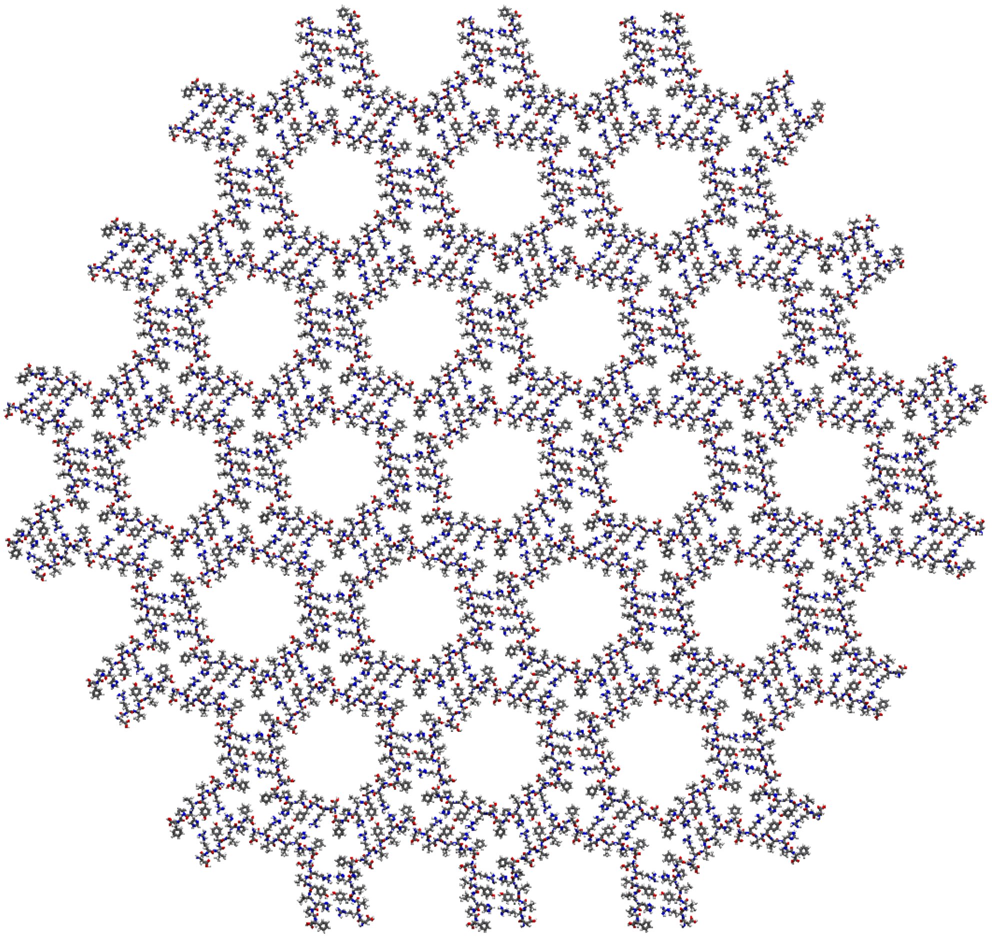

Supplementary Figure 6: MD optimized structure of large area of the network. The simulation is stable over a long run. The peptides at the rim of the structure are not fixed, thus they show more deviation from the optimal structure within the network. Conformational variation can be observed within the network as this simulation did not apply periodic boundary conditions.

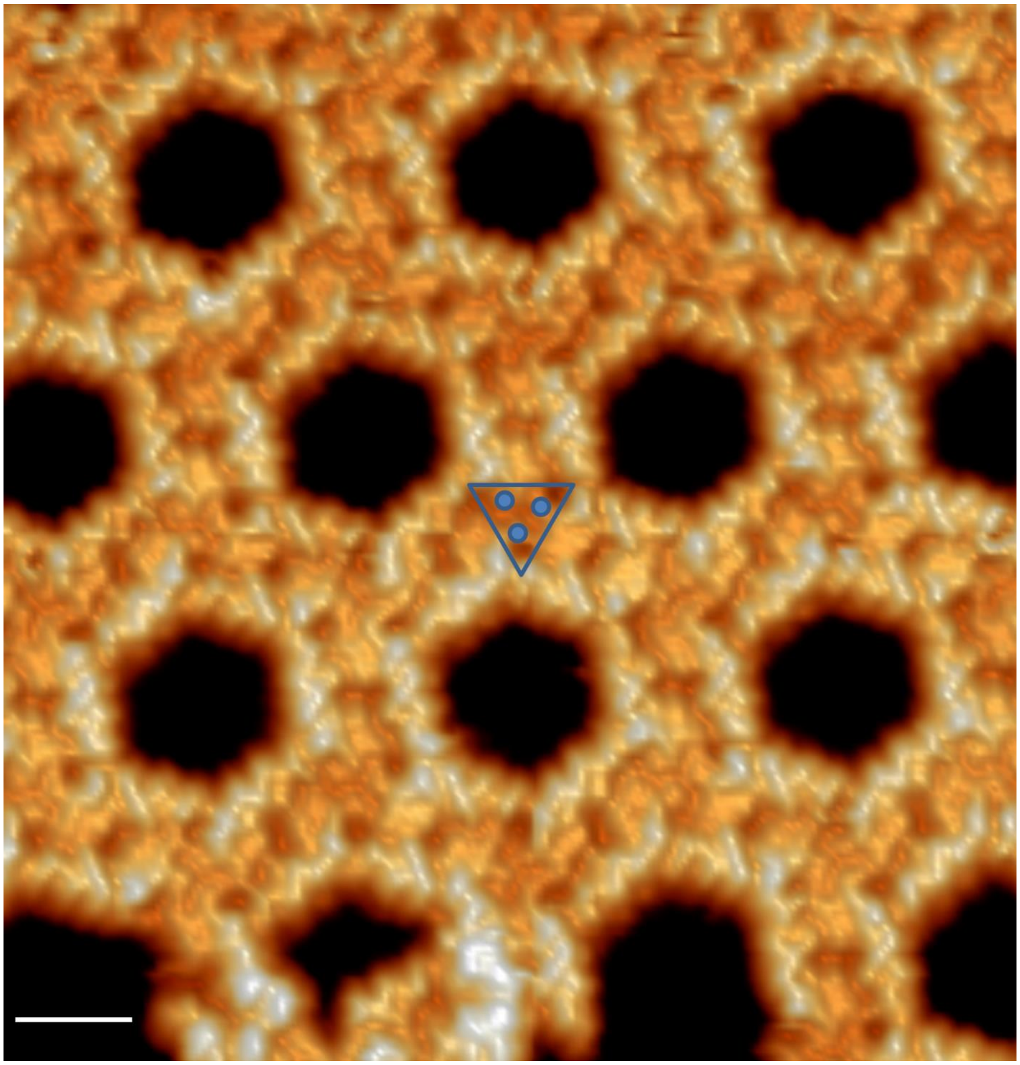

Supplementary Figure 7: High-resolution STM images of At-II at 40K. The backbone of the peptide shows the highest contrast at the edge of the pores. At the vertex, the three protrusions due to the Phe8 are visible and marked with blue circles in the central vertex. The areas with low intensity within the vertex are connected by the blue triangle. The scale bar is 2 nm.

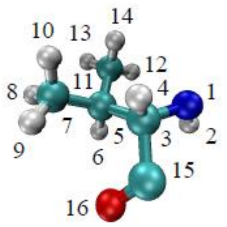

Supplementary Figure 8: Typical conformation of the Val residue. Carbon, oxygen, nitrogen and hydrogen atoms are shown in green, red, blue and white, respectively. Equations (1)-(8) and tables 1-4 provide a direct determination of the intramolecular interaction potential, where an index i has been assigned to each atom.

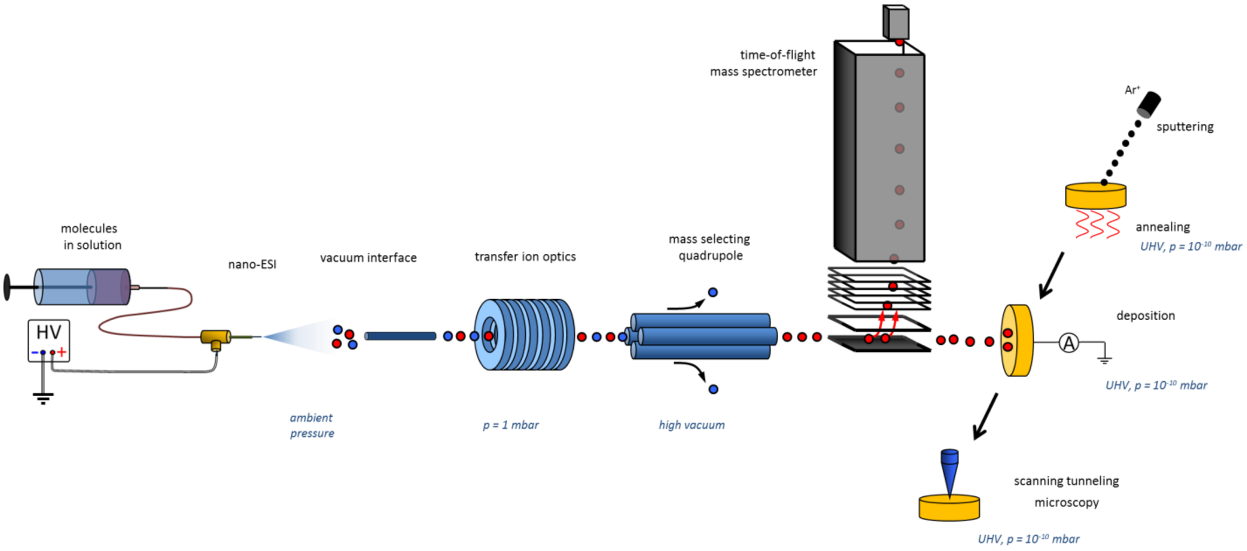

Supplementary Figure 9: Scheme of the electrospray ion beam deposition setup and procedure. Molecular ions are created by the nano-ESI source and interface (left). The ions are transferred by ion optics to a sample in UHV. A quadrupole mass filter and a TOF mass spectrometer ensure the chemical composition of the ion beam. In a parallel process, a metal surface is cleaned by several sputtering/annealing cycles in a separate UHV-chamber. The clean sample is transferred to the deposition chamber. After deposition, the sample is moved to the scanning tunneling microscope without breaking the vacuum.

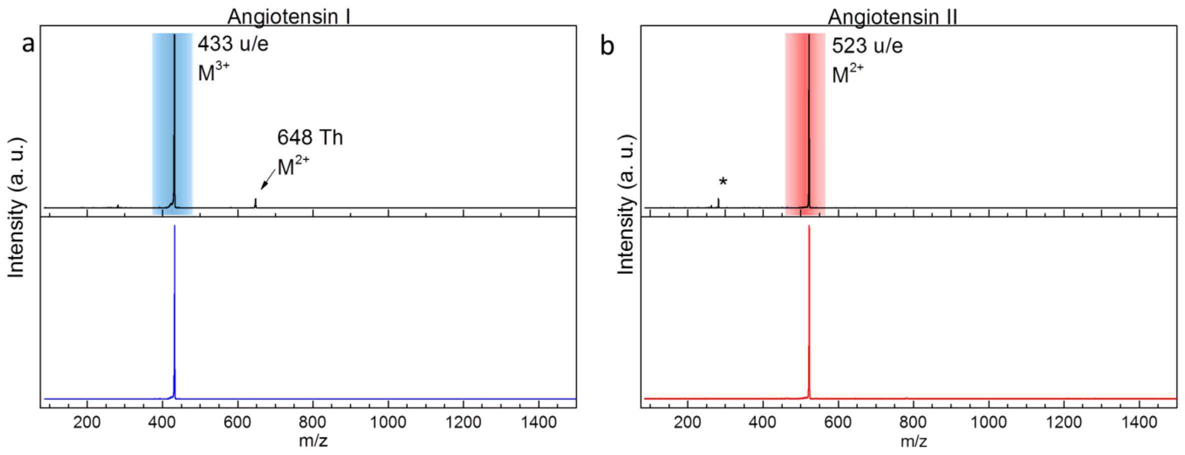

Supplementary Figure 10: Time-of-flight mass spectra with the mass filter window indicated. a, Mass spectra of **At-I** before and after mass selection. The most intense peak is found for the triply protonated peptide at m/z=433 u/e. **b,** Mass spectra of **At-II** before and after mass selection. The most intense peak at m/z=524 u/e corresponds to the doubly protonated peptide, the peak indicated by * marks contamination.

Supplementary Tables

Index i	z_i	σ_{ii} / nm	ϵ_{ii} mol kJ^{-1}	Element symbol
1	-0.5	0.325	0.711	N
2	0.3	0	0	H
3	0.14	0.35	0.276	C
4	0.06	0.25	0.126	H
5	-0.06	0.25	0.276	C
6	0.06	0.25	0.126	H
7	-0.18	0.25	0.276	C
8	0.06	0.25	0.126	H
9	0.06	0.25	0.126	H
10	0.06	0.25	0.126	H
11	-0.18	0.25	0.276	C
12	0.06	0.25	0.126	H
13	0.06	0.25	0.126	H
14	0.06	0.25	0.126	H
15	0.5	0.375	0.439	C
16	-0.5	0.296	0.879	O

Supplementary Table 1: Parameters of the Lennard-Jones potential and the Coulomb potential used for the Val residue.

Index i	Index j	b_{ii} / nm	$k_{ij}^{(b)}$ mol kJ^{-1}
1	2	0.1010	363171.2
1	3	0.1449	282001.6
3	4	0.1090	284512.0
3	5	0.1529	224262.4
3	15	0.1522	265265.6
5	6	0.1090	284512.0
5	7	0.1529	224262.4
5	11	0.1529	224262.4
7	8	0.1090	284512.0
7	9	0.1090	284512.0
7	10	0.1090	284512.0
11	12	0.1090	284512.0
11	13	0.1090	284512.0
11	14	0.1090	284512.0
15	16	0.1229	476976.0

Supplementary Table 2: Parameters of the bond stretching potential used for the Val residue.

Index i	Index j	Index k	$\vartheta_{ijk}^{(0)}$ /nm	$k_{ijk}^{(a)}$ mol kJ^{-1} rad^{-2}
2	1	3	118.4	317.984
1	3	4	109.5	292.880
1	3	5	109.5	669.440
1	3	15	110.1	527.184
4	3	5	110.7	313.800
4	3	15	109.5	292.800
5	3	15	111.1	527.184
3	5	6	110.7	313.800
3	5	7	112.7	488.273
3	5	11	112.7	488.273
6	5	7	110.7	313.800
6	5	11	110.7	313.800
7	5	11	112.7	488.273
5	7	8	110.7	313.800
5	7	9	110.7	313.800
5	7	10	110.7	313.800
8	7	9	107.8	276.144
8	7	10	107.8	276.144
9	7	10	107.8	276.144
5	11	12	110.7	313.800
5	11	13	110.7	313.800
5	11	14	110.7	313.800
12	11	13	107.8	276.144
12	11	14	107.8	276.144
13	11	14	107.8	276.144
3	15	16	120.4	669.440

Supplementary Table 3: Parameters of the bond angle vibration potential used for the Val residue.

Index i	Index j	Index k	Index l	$C_0^{(ijkl)}$ mol kJ^{-1}	$C_1^{(ijkl)}$ mol kJ^{-1}	$C_2^{(ijkl)}$ mol kJ^{-1}	$C_3^{(ijkl)}$ mol kJ^{-1}
2	1	3	4	0	0	0	0
2	1	3	5	0	0	0	0
2	1	3	15	0	0	0	0
1	3	5	7	4.50199	0.78241	-1.60247	-3.68192
1	3	5	11	4.50199	0.78241	-1.60247	-3.68192
15	3	5	7	0.42259	2.70705	-3.12964	0
15	3	5	11	0.42259	2.70705	-3.12964	0
1	3	5	6	0.97069	2.91206	0	-3.88275
4	3	5	6	0.62760	1.88280	0	-2.51040
4	3	5	7	0.62760	1.88280	0	-2.51040
4	3	5	11	0.62760	1.88280	0	-2.51040
15	3	5	6	-0.15899	-0.47698	0	0.63596
1	3	15	16	0	0	0	0
4	3	15	16	0	0	0	0
5	3	15	16	0	0	0	0
3	5	7	8	0.62760	1.88280	0	-2.51040
3	5	7	9	0.62760	1.88280	0	-2.51040
3	5	7	10	0.62760	1.88280	0	-2.51040
6	5	7	8	0.62760	1.88280	0	-2.51040
6	5	7	9	0.62760	1.88280	0	-2.51040
6	5	7	10	0.62760	1.88280	0	-2.51040
11	5	7	8	0.62760	1.88280	0	-2.51040
11	5	7	9	0.62760	1.88280	0	-2.51040
11	5	7	10	0.62760	1.88280	0	-2.51040
3	5	11	12	0.62760	1.88280	0	-2.51040
3	5	11	13	0.62760	1.88280	0	-2.51040
3	5	11	14	0.62760	1.88280	0	-2.51040
6	5	11	12	0.62760	1.88280	0	-2.51040
6	5	11	13	0.62760	1.88280	0	-2.51040
6	5	11	14	0.62760	1.88280	0	-2.51040
7	5	11	12	0.62760	1.88280	0	-2.51040
7	5	11	13	0.62760	1.88280	0	-2.51040
7	5	11	14	0.62760	1.88280	0	-2.51040

Supplementary Table 4: Parameters of the Ryccaert-Belleman dihedral potential used for the Val residue.

Supplementary Notes

Supplementary Note 1: STM Characterization of AT-I

Survey Images of At-I

After deposition and subsequent cooling to 40 K, the **At-I** molecules arrange on the Au(111) surface as depicted in the STM images of in Supplementary Fig. 1. Two structures **A** (chain of dimers) and **B** (compact) can be found on the surface, however, there is no preferred alignment with the Au(111) reconstruction, i.e. the substrates principal directions.

Additional molecules attached at the rim of the structure **B** can be frequently observed. The attachment site is exclusively the row of low contrast, as indicated by an arrow in Supplementary Fig. 1c. This agrees very well with our model, in which the polar C-terminal is exposed at this position and hence is available for further interaction. In contrast, structure **A** does not show additional attachment to the dimer chains due to better passivation of the structure by nonpolar moieties, as demonstrated in Fig. 1d. Individual molecular rotors anchored at the elbow site of the Au(111) reconstruction suggest that the molecules would still be mobile if not fixed within an assembly or at a pinning site.

Details of Structural Assignment and Comparison to Simulated STM Images

In order to verify the correctness of the assignment, as shown in Fig. 1a-f, it is very helpful to consider single peptides. However, due to the high mobility and stable aggregation, it is difficult to find isolated molecules of **At-I** on Au(111) that can clearly be identified as such. Thus, we focus on defects to infer the shape and structure of the individual peptide.

In Supplementary Fig. 2a, a dimer chain of structure **A** is shown with two complete dimers and one defect dimer at the top. The intact dimers are characterized by a double row of 4 features with two features in between. While the C_2 symmetry confirms that this structure is a dimer, it is unclear how to assign the single molecule within the dimer. The assignment can only be attained by considering the defect dimer structure at the top of the dimer row in Supplementary Fig. 2a. Here, half of the dimer is in place, presenting an appearance that agrees very well with half of the intact dimer. Thus, we assign it to a single peptide.

With this, it is now possible to assign the orientation of the monomers within the dimer. This information is used to validate the final MD model of the dimer as shown in the main manuscript (Fig.

1d) and in Supplementary Fig. 2b. As STM images convolute local density of electronic states with height information, the molecular structures obtained by MD cannot directly be compared to STM data. To confirm and compare the results, we further computed STM images based on the MD structures.

The simulated STM image (Supplementary Fig.S2c) reproduces the key features of the measured structures very well: Two pronounced features inside each dimer are attributed to two aromatic functional groups (Tyr4 and His6). Additionally, the rim of the assembly is pronounced due to their height, while the inter-dimer region shows low signal as the C-terminal as well as Asp1, Arg2 and Leu10 have very low electron density within the range of 2 eV from the HOMO.

From the symmetry of the structure **B**, we deduce that the peptides are stacked in a parallel manner and adjacent stacks are rotated by 180 °, as discussed in the main manuscript. At the edge of these stacks, the characteristic shape of the individual molecule of structure **A** can also be found, as depicted in Supplementary Fig. 3a. Size and shape are very similar, but the intensity within the molecule is changed compared to structure **A**. This might be due to slight conformational variance which can be reproduced in the MD optimized structures (Supplementary Fig. 3b and Fig 1 in the main manuscript), such as the position of the Arg2 residue influencing the relative distance to the substrate.

In the simulated STM images for structure B (Supplementary Fig. 3c), the contrast at the two different interfaces of the molecular rows is well reproduced. The interface at the C-terminal shows a wide region with very little intensity. Only the aromatic rings of the His9 show signal within this interface accounting for the zipper-like structure that can also be observed in the experimental STM image (Fig. 1f). At the other interface including the N-terminal, a row of high intensity is observed experimentally. In the simulated STM image, the intensity is carried by the N-terminal and the first peptide bond.

Supplementary Note 2: STM Characterization of AT-II

Survey Image of At II

After deposition of **At-II**, the molecules are still mobile on the surface. They can only be imaged by STM upon cooling. During cool down a large number of rotors is present as well as streaks in the STM image, indicative of highly mobile molecules, as demonstrated in Supplementary Fig 4a. After cooling to 40 K, large networks are obtained nearly exclusively starting on the terraces rather than on the step edges, shown in Supplementary Fig. 4b. Peptides that are not assembled in the network are partially mobile and can be seen as rotors (arrows in Supplementary Fig. 4b) anchored at the elbow sites of the Au(111) reconstruction. These are probable candidates for the seeds of the assemblies.

Detailed Discussion of At-II Single Peptides at Defect Sites

By giving voltage pulses from the tip to the sample into the network, it is possible to disassemble the network and get additional insight into single peptide structures. In the upper part of Supplementary Fig. 5a, we observe the dimers that are still assembled into the network of **At-II**. Next to it, we can find single peptides (marked with a green circle). They have a characteristic shape in which the lower part is thinner and low in intensity, whereas the wider part has several intense features. We find the same shape for the dimers and therefore conclude that the conformation does not change significantly.

When the individual peptide is stabilized at defects within the network, one feature for nearly every polar amino acid is observed. This is shown in Supplementary Figs. 5b and 5c. The overall shape of the molecule is very well reproduced by the simulated STM image of the MD optimized structure, shown in Supplementary Fig. 5d: in general, the backbone gives the highest signal due to the electron rich peptide bonds and its height. At the N-terminal, the shape appears thinner as the Arg2 and Asp1 do not carry much electron density and thus do not give much signal. In contrast, the functional groups close to the C terminal are intense due to the electron rich groups of His6 and Phe8.

High-Resolution STM Image of At II

In the high resolution image of the At-II network (Supplementary Fig. 7), one can clearly observe the high degree of order of the network. The individual molecule is concealed by the intimate interaction with its neighboring molecules. The conformation variability might be huge for single peptides, but the crystallization in the network reduces this conformational space to only one preferred conformation within the resolution of the STM.

Supplementary Methods

Rationale and Methodology of the Data Analysis and Interpretation

Due to the large size and complexity of the molecular system treated here, it is impossible to deduce the molecular structure of the observed assemblies directly from the STM images. Therefore, a synergy of theory (MD and DFT) and experiment (STM and MS) is pivotal to understand new peptide structures formed on the surface. Here, we describe the rationale and methodology of this process. In the following sections, we will discuss each point in more detail.

1) We can make sure that we have only intact peptides in the ion beam by **monitoring the mass spectrum**. Via a **mass filtered deposition**, we cut off all possible fragments or unspecified species and make sure that we only deposit the purified molecular ion.

2) For the deposition, we intentionally **decelerate the molecular ions** to a kinetic energy of 10 eV to avoid fragmentation of the molecules upon landing and thus ensure the adsorption of only intact peptides. We have shown soft landing for a number of molecules, such as the 104 AA protein Cytochrome C[1], or dyes Rhodamine 6G[2] and N3[3], and there are several examples in literature also including peptides[4]. We are thus certain that for the deposition at low energy, the molecules are **landed intact on the metal surface.**

3) The subsequent characterization of the peptides on the surface is performed with **STM.** This technique is most suited for **the high-resolution investigation in real space**. Here, we observe structures that fit to the size of the molecules. However, due to the complexity of the molecules, atomic resolution is not possible. Moreover, due to the convolution of local electron density and height in the STM images, the hybridization of molecular orbitals with the metal substrate and the closed interlocked molecules in the assemblies, the structures cannot be interpreted unambiguously and thus need the support of theory.

4) The first step of the modelling is **the investigation of the landing process** of the peptides on the surface with **MD simulations**. From these simulations, we obtain initial adsorption conformations for peptides on the surface.

5) We use these quasi-2D conformations as starting configuration to model the self-assembled structures we observe by STM. The information from STM, such as symmetry, morphology, and defect sites are used to validate the final model. MD modelling allows to find the position of the atoms to understand the interactions in detail.

6) In order to be able to compare the MD modelled structures to the experimental images, we **simulate the STM image** (electron density within 2 eV from HOMO of the molecule) **by DFT**. Due to the size and complexity of the system, we approximate the situation by simulating the STM image without taking the surface into account. To validate the optimized structure, the simulated STM image needs to agree with the experimental STM image.

Sample Preparation

Soft-Landing Electrospray Ion Beam Deposition

Solutions of angiotensin I and II (Sigma Aldrich A9650 and A9525, respectively) for mass spectrometry and subsequent deposition were prepared by dissolving the peptide in a water-ethanol 1:1 mixture with approx. 0.1% formic acid to result in a concentration of 10^{-4} mol/L. Positively charged gas phase ions were generated by a nano-electrospray source with 3-4 kV applied at the emitter and a solution flow rate of 20-25 µL/hr. The deposition apparatus consists of six differentially pumped vacuum chambers reducing the pressure from ambient to 10^{-10} mbar. After transfer into vacuum, the ions pass several ion optics for collimation and transmission, including a quadrupole mass-filter, before reaching the TOF mass spectrometer and further downstream the deposition sample holder (see Supplementary Fig. 9).

After taking a survey spectrum of a beam containing all ions, the mass-filter is tuned to transmit only the required ion species, that is *m/z*=433 u/e for the triple-protonated At-I ion and *m/z*=524 u/e for the doubly charged At-II ion, as shown in Supplementary Fig.10.

For the deposition on the Au(111) sample, which is cleaned with several sputter and annealing cycles prior to deposition, the ion beam is further guided through two following apertures leading to the deposition chamber at a pressure of 10^{-10} mbar. A positive voltage applied to the sample decelerates the ions to 10 eV for soft landing. The ion current of the sample is monitored online using electrometers (Keithley 616/617). Approx. one hundred picoamperes (pA) of mass selected ion current reach the sample position. Thus a deposition of a charge of 30-60 pAh usually takes less than one hour.

After deposition, the sample is transferred in-situ to a variable temperature STM (Omicron VT-STM, Omicron Nanotechnology GmbH, Germany), and subsequently cooled down to 40 K for imaging.

Mass Spectrometry and Mass Selection

Time-of-flight mass spectra of the beam were obtained before and after mass selection as shown in Fig. 9 upper and lower panel, respectively. For both, **At-I** (Supplementary Fig. 10a) and **At-II** (Supplementary Fig. 10b), the initial spectra are very clean as purified peptide salts were used for the solution. Nonetheless, to ensure high purity deposition and suppress chemical noise for instance in the form of solvent clusters, the beams were mass filtered using the rf-quadrupole in the third pumping stage.

For the ion beam of **At-I**, the +3 charge state has the highest intensity and was thus mass selected for deposition, as shown in Fig. Supplementary 9a. For **At-II**, the +2 charge state has the highest abundance while charge state +1 is barely visible on the linear scale. Therefore, we chose to deposit charge state +2 to yield a high current for the deposition. The distribution of intensity shifts between the charge states as a function of the pH value. The doubly protonated species gains in intensity at lower concentration of formic acid, while the +1 charge state is not observed in these experiments for angiotensin I. It is of no consequence which of the protonated species is deposited since the attached protons rapidly dissipate after surface deposition and leave the same neutral peptide species.

Modelling: Molecular Dynamics

Potentials and Force Fields

All MD simulations were performed using GROMACS software[5] (see Ref [5] and references therein) together with OPLS-AA force field[6-9]. The energy function for the simulation comprises non-bonded interactions described by Coulomb and van-der-Waals potentials as well as bonded interactions such as bond stretch, bond angle and dihedral terms which are evaluated according to the following Eqs. (1) - (8):

- The Lennard-Jones potential V_{LJ} is a combination of attractive van-der-Waals interaction and the repulsive interaction due to the Pauli exclusion principle:

$$V_{LJ}(r_{ij}) = 4\epsilon_{ij}\left(\left(\frac{\sigma_{ij}}{r_{ij}}\right)^{12} - \left(\frac{\sigma_{ij}}{r_{ij}}\right)^{6}\right), \tag{1}$$

With the geometric averages

$$\sigma_{ij} = \sqrt{\sigma_{ii}\sigma_{jj}} \quad , \tag{2}$$

$$\epsilon_{ij} = \sqrt{\epsilon_{ii}\epsilon_{jj}} \quad . \tag{3}$$

Here it proves convenient to assign an index i to each atom of the system, where the system consists of all deposited peptides and the Au(111) substrate. The indices $i \in [1, N_{Pep}]$ belong to the atoms of the deposited peptides and the indices $i \in [N_{Pep} + 1, N_{Pep} + N_{Cu}]$ belong to the

copper atoms, where N_{Pep} and N_{cu} are the total numbers of peptide and copper atoms, respectively. Moreover, r_{ij} is the center-to-center distance between to atoms of type *i* and *j*.

- The Coulomb potential V_C between two atoms with valency z_i and z_j is given by

$$V_{\mathrm{C}}(r_{ij}) = \frac{e^2}{4\pi\epsilon_0}\frac{z_i z_j}{r_{ij}} \ , \tag{4}$$

with

$$\frac{e^2}{4\pi\epsilon_0} = 138.935 \text{ kJ nm mol}^{-1} \ . \tag{5}$$

The bond stretching potential between two covalently bonded atoms of type *i* and *j* is represented by the harmonic potential

$$V_{\mathrm{b}}(r_{ij}) = \frac{1}{2}k_{ij}^{(b)}(r_{ij} - b_{ij})^2 \ , \tag{6}$$

where b_{ij} denotes the bond length for which the potential V_{b} exhibits a minimum as a function of the center-to-center distance r_{ij} between the two atoms.

- The bond-angle vibration potential between covalently atoms of type *i*, j and *k* is given by

$$V_{\mathrm{a}}(\vartheta_{ijk}) = \frac{1}{2}k_{ijk}^{(\mathrm{a})}(\vartheta_{ijk} - \vartheta_{ijk}^{(0)})^2 \ , \tag{7}$$

where $\vartheta_{ijk}^{(0)}$ denotes the angle for which the potential V_a exhibits a minimum as a function of the angle ϑ_{ijk} formed by the lines along r_{ij} and r_{jk}. According to this notation, the atom of type *j* is covalently bonded to the atoms of type *i* and *k*.

- The intramolecular Ryckaert-Belleman dihedral potential between atoms of type *i, j, k* and *l* reads

$$V_{\mathrm{rb}}(\varphi_{ijkl}) = \sum_{n=0}^{3} C_n^{(ijkl)} \cos^n(\varphi_{ijkl} - 180°) \ , \tag{8}$$

Where the dihedral angle φ_{ijkl} is defined as the angle between the planes spanned by the atoms of type *i, j, k* and *j, k, l*. Hence, three successive chemical bonds characterized by the successive center-to-center distances r_{ij}, r_{jk} and r_{kl} are considered. Upon varying φ_{ijkl}, only the distance r_{il} between the atoms of type *i* and *l* is changed. The other interatomic bond lengths and angles do not depend on φ_{ijkl}.

Description of the Substrate

During the MD simulations, the positions of the gold atoms were frozen at their crystallographic values. Moreover, the OPLS-AA force field has been extended by using the GoIP force field[10,11] in order to take into account the interaction between the gold surface atoms and the atoms of the peptides. Within this approach a dynamical ensemble of dipolar rods carrying two charges of valencies 0.3 and -0.3, which are separated at a distance of 0.7 nm, is used to capture image charge effects, while the Lennard-Jones parameters in Eqs. (2) and (3) are given by σ_{AuAu} = 0.32 nm and ϵ_{AuAu} = 0.65 kJ mol^{-1} for gold atoms, i.e. $\sigma_{jj} = \sigma_{\mathrm{Au,Au}}$ and $\epsilon_{jj} = \epsilon_{\mathrm{Au,Au}}\ \forall\, j \in\ [N_{\mathrm{Pep}} + 1,\ N_{\mathrm{Pep}} +\ N_{\mathrm{Cu}}]$. Hence, the Lennard-Jones parameters for the interaction between a peptide atom of type $i \in\ [1,\ N_{Pep}]$ and a gold atom labelled with the index $j \in\ [N_{\mathrm{Pep}} + 1,\ N_{\mathrm{Pep}} +\ N_{\mathrm{Cu}}]$ are evaluated according to Eqs. (2) and (3) as $\sigma_{ij} = \sqrt{\sigma_{ii}\sigma_{\mathrm{Au,Au}}}$ and $\epsilon_{ij} = \sqrt{\epsilon_{ii}\epsilon_{\mathrm{Au,Au}}}$. This theoretical concept has already been applied to various systems[12-16].

Modelling the Landing Process

As a starting point of the MD simulations, the charged peptides were relaxed in vacuum resulting in initial three-dimensional gas phase structures. In the gas phase, due to excess of charge, no aggregation happens. For comparison we note that corresponding three-dimensional solution phase structures of both **At-I** and **At-II** have been studied earlier using NMR spectroscopy together with restrained energy refinement[17]. Subsequently, we added electric fields perpendicular to the sample to match the experimental conditions. Upon landing, the peptides relax into mobile, quasi two-dimensional configurations which are rather independent of the initial gas phase structures. This result applies specifically to the case of the Au(111) surface due the weaker interactions between the peptides and the substrate as compared with, e.g, a Cu(100) surface. For the latter surface we have previously found a dependence of the final configuration of the protein cytochrome c on the initial orientation of the protein in the gas phase due to a reduced protein mobility on the strongly interacting Cu(100) surface[18]. In summary, during the collision of the peptides with the Au(111) surface, the initial three-dimensional gas phase conformations are reduced into two-dimensional adsorption geometries, i.e., all atoms of the peptides are located inside a layer of thickness 0.4 nm above the substrate. Additional MD simulations of **At-I** and **At-II** adsorption on surfaces with weaker peptide-surface interactions exhibited an increase of the aforementioned layer thickness with decreasing peptide-surface interaction strength leading ultimately to three-dimensional peptide structures. In general, the peptide configurations have to be considered as representatives of statistical ensembles due to the fact that peptides can take up a huge number of conformations by the rotation of chemical bonds in accordance with the Ryckaert-Belleman dihedral potential (see Eq. (8)).

Modelling the Assembly

In order to model the self-assembly, we use the neutral molecular configurations of a single peptide resulting from the landing process as starting configuration. To overcome the long time scale necessary for the collective dynamic to form the self-assembly, we use starting positions that agree with the constraint given by the experimental observations (good initial guesses), such as symmetry, morphology, and defect sites, as well as simulated annealing. Once a stable structure is formed, the refinement is modelled in real time. As the peptides can take up a huge number of conformations, we also find some variability in the structures as can be seen in the overview image of the MD optimized structure of the honeycomb network (Fig S6). However, these variations are within the limit of the STM resolution and thus do not hinder the structural assignment of the STM measurements.

In order to illustrate typical values of the model parameters in Eqs. (1) - (8), we focus on the amino acid valine (Val) which is a residue of both **At-I** and **At-II** according to Fig. 2 of the main manuscript. Supplementary Fig. 8 displays a typical conformation of the Val residue using a ball-and-stick representation. An index has been assigned to each atom such that such the actual values of the parameters z_i, σ_{ii}, ϵ_{ii} , b_{ij} , $k_{ij}^{(b)}$, $\vartheta_{ijk}^{(0)}$, $k_{ijk}^{(a)}$, $C_n^{(ijkl)}$ with $i, j, k, l \in [1, 16]$, $i \neq j \neq k \neq$ l and $n = 0, 1, 2, 3$ can be deduced from Supplementary Tab. 1 - 4. The strong harmonic bond stretching and bond-angle vibration potentials do allow only for slight fluctuations of bond lengths and bond angles around equilibrium values. According to Supplementary Tab. 2, the C-C bond lengths are the largest bond lengths that exist for the Val residue, while the N-H bond length is the shortest one. From Supplementary Tab. 1, it is apparent that the partial charges of the Val residue are in particular located at the atoms which contribute to the peptide bonds with the two neighboring amino acids (see Fig. 2 of the main manuscript). According to Supplementary Tab. 4, the heights of the energy barriers between different conformations of four consecutive bonded atoms are comparable to the thermal energy ($k_B T = 2.49$ kJ mol^{-1} for T = 300 K) leading to thermal induced conformational changes of peptides.

Supplementary References

1 Deng, Z. *et al.* A close look at proteins: Submolecular resolution of two- and three-dimensionally folded cytochrome c at surfaces. *Nano Lett.* **12**, 2452-2458 (2012).

2 Rauschenbach, S. *et al.* Electrospray ion beam deposition: Soft-landing and fragmentation of functional molecules at solid surfaces. *ACS Nano* **3**, 2901-2910 (2009).

3 Kley, C. S. *et al.* Atomic-scale observation of multiconformational binding and energy level alignment of ruthenium-based photosensitizers on TiO2 anatase. *Nano Lett.* **14**, 563-569 (2014).

4 Laskin, J., Wang, P. & Hadjar, O. Soft-landing of peptide ions onto self-assembled monolayer surfaces: an overview. *Phys. Chem. Chem. Phys.* **10**, 1079-1090 (2008).

5 van der Spoel, D., Marklund, E. G., Larsson, D. S. D. & Caleman, C. Proteins, lipids, and water in the gas phase. *Macromol. Biosci.* **11**, 50-59 (2011).

6 Jorgensen, W. L. & Tirado-Rives, J. The OPLS [optimized potentials for liquid simulations] potential functions for proteins, energy minimizations for crystals of cyclic peptides and crambin. *J. Am. Chem. Soc.* **110**, 1657-1666 (1988).

7 Kaminski, G., Duffy, E. M., Matsui, T. & Jorgensen, W. L. Free energies of hydration and pure liquid properties of hydrocarbons from the OPLS all-atom model. *J. Phys. Chem.* **98**, 13077-13082 (1994).

8 Jorgensen, W. L., Maxwell, D. S. & Tirado-Rives, J. Development and testing of the OPLS all-atom force field on conformational energetics and properties of organic liquids. *J. Am. Chem. Soc.* **118**, 11225-11236 (1996).

9 Jorgensen, W. L. & Tirado-Rives, J. Potential energy functions for atomic-level simulations of water and organic and biomolecular systems. *Proc. Natl. Acad. Sci. USA* **102**, 6665-6670 (2005).

10 Iori, F. & Corni, S. Including image charge effects in the molecular dynamics simulations of molecules on metal surfaces. *J. Comput. Chem.* **29**, 1656-1666 (2008).

11 Iori, F., Di Felice, R., Molinari, E. & Corni, S. GolP: An atomistic force-field to describe the interaction of proteins with Au(111) surfaces in water. *J. Comput. Chem.* **30**, 1465-1476 (2009).

12 Hoefling, M., Iori, F., Corni, S. & Gottschalk, K.-E. The conformations of amino acids on a gold(111) surface. *ChemPhysChem* **11**, 1763-1767 (2010).

13 Hoefling, M., Iori, F., Corni, S. & Gottschalk, K.-E. Interaction of amino acids with the Au(111) surface: Adsorption free energies from molecular dynamics simulations. *Langmuir* **26**, 8347-8351 (2010).

14 Hoefling, M., Monti, S., Corni, S. & Gottschalk, K. E. Interaction of beta-sheet folds with a gold surface. *Plos One* **6** (2011).

15 Barone, V., Casarin, M., Forrer, D., Monti, S. & Prampolini, G. Molecular dynamics simulations of the self-assembly of tetraphenylporphyrin-based monolayers and bilayers at a silver interface. *J. Phys. Chem. C* **115**, 18434-18444 (2011).

16 Wright, L. B., Rodger, P. M., Corni, S. & Walsh, T. R. GolP-CHARMM: First-principles based force fields for the interaction of proteins with Au(111) and Au(100). *J. Chem. Theory Comput.* **9**, 1616-1630 (2013).

17 Spyroulias, G. A. *et al.* Comparison of the solution structures of angiotensin I & II. *Eur. J. Biochem.* **270**, 2163-2173 (2003).

18 Rinke, G. *et al.* Active conformation control of unfolded proteins by hyperthermal collision with a metal surface. *Nano Lett.* **14**, 5609-5615 (2014).
